# Sjögren Syndrome Complicated with Cystic Lung Disease and Pulmonary Amyloidosis

**DOI:** 10.1155/2018/7475242

**Published:** 2018-03-31

**Authors:** Koichiro Takahashi, Hironori Sadamatsu, Shinsuke Ogusu, Kazutoshi Komiya, Tomomi Nakamura, Shinya Kimura, Naoko Sueoka-Aragane

**Affiliations:** Division of Hematology, Respiratory Medicine and Oncology, Department of Internal Medicine, Faculty of Medicine, Saga University, 5-1-1 Nabeshima, Saga 849-8501, Japan

## Abstract

A 72-year-old Japanese woman was noted to have multiple cystic lung shadows and infiltrates on chest radiography and computed tomography (CT). She complained of dryness of the mouth and eyes, but she did not have respiratory symptoms, such as cough, sputum production, and dyspnea. Her laboratory findings showed high titers of anti-SSA/Ro and anti-SSB/La antibodies. Surgical lung biopsy was performed and demonstrated pathologic findings of amyloid light-chain deposition and bronchiolitis with lymphocytic infiltration. Taken altogether, she was diagnosed as Sjögren syndrome with bronchiolitis and pulmonary amyloidosis. Since then, she has been carefully followed up without treatment. After 6 years, the cystic lung lesions on CT gradually enlarged and increased in number, but she remained to have no respiratory symptoms and no manifestations of lymphoma. Here, we report a rare case of Sjögren syndrome complicated with cystic lung disease and pulmonary amyloidosis.

## 1. Introduction

Sjögren syndrome is associated with various respiratory complications; its most common manifestations are interstitial lung disease and tracheobronchial disease [1], but less common manifestations, such as cystic lung lesions and pulmonary amyloidosis, have been reported [2, 3].

Amyloidosis is characterized by extracellular deposition of amyloid in various tissues; the amyloid proteins are fibrillary materials that are mainly classified into about 30 types of proteins [4]. In amyloid light-chain (AL) amyloidosis, the tissue deposits comprise immunoglobulin light chains. Amyloid localized to the respiratory system is classified as tracheobronchial, mediastinal or hilar lymphadenopathy, pleural effusion, and lung parenchymal [5, 6]. When the lung parenchyma is affected, nodules, infiltrates, and interstitial depositions are observed [7]. Interestingly, in the present case, the cystic lung lesions on chest computed tomography (CT) gradually enlarged and increased in number over a 6-year period. The present case is considered to be valuable because it presented evaluation of the progression of the cystic changes in the lung over time. We here report a case of Sjögren syndrome with cystic lung disease and pulmonary amyloidosis.

## 2. Case Presentation

A 72-year-old Japanese woman visited our hospital because she was noted to have an abnormality on chest radiography upon routine health examination. She recalled having the sensation of dryness of the mouth and eyes for 1 year, but she did not have fever and any respiratory symptoms, such as cough, sputum production, or dyspnea. She had never smoked, and past medical history was significant for uterine fibroid.

**Figure 1:**
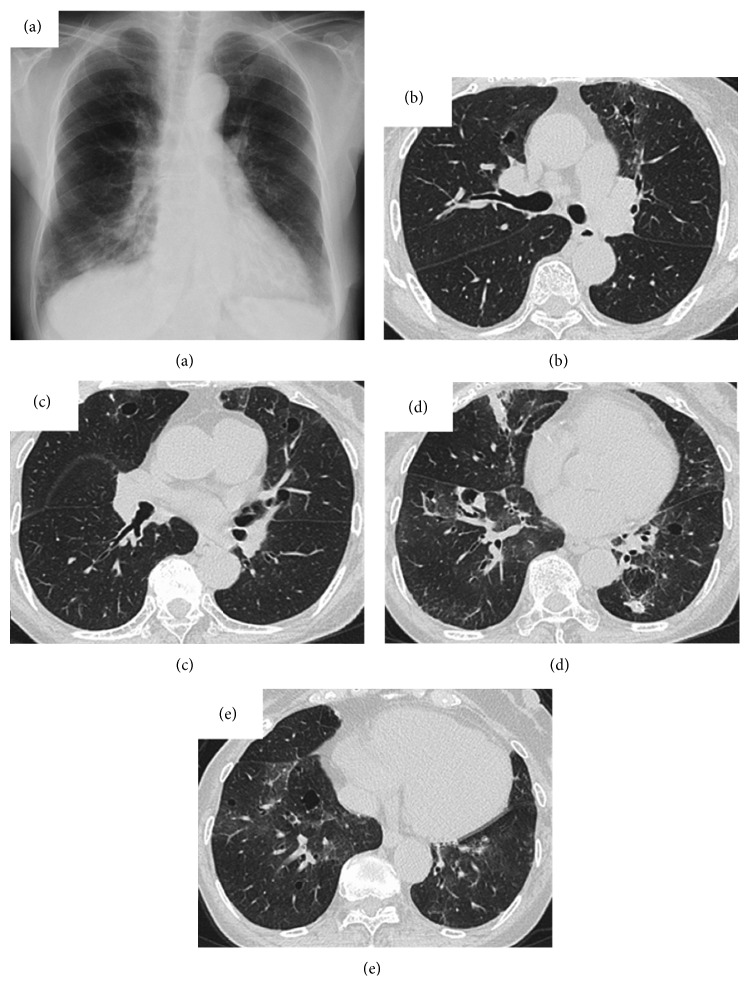
Chest radiograph and CT findings in a 72-year-old woman with Sjögren syndrome and pulmonary amyloidosis. (a) Chest radiograph shows infiltrates in the right lower lung field and ground-glass shadows in the bilateral lower lung fields. Chest CT shows (b, c) multiple cystic lung shadows in the bilateral upper lobes, (d) infiltrates in the right middle lobe and a nodular shadow in the left lower lobe, and (e) ground-glass shadows in the bilateral lower lobes. CT, computed tomography.

Physical examination showed blood pressure of 126/78 mmHg, heart rate of 72 beats per minute, respiratory rate of 16 per minute, body temperature of 36.3°C, and the absence of crackles on auscultation. Chest radiography showed infiltrates on the right lower lung field, and chest CT showed multiple infiltrates and cystic lesions in the bilateral lung (Figure 1). Laboratory findings showed hemoglobin 12.8 g/dL, white blood cell count 5,000/*μ*L, platelet count 24.6 × 104/*μ*L, C-reactive protein (CRP) 0.04 mg/dL, anti-nuclear antibody ×160, anti-SSA antibody >500 U/mL, and anti-SSB antibody 101 U/L. The total protein in plasma (8.6 g/dL) comprised 40.7% albumin, 2.7% alpha-1, 7.6% alpha-2, 7.4% beta, and 41.6% gamma-globulin, including immunoglobulin (Ig) G at 2,101 mg/dL, IgG4 at 19.6 mg/dL, and IgA at 999 mg/dL. Serum immunoelectrophoresis showed monoclonal M-protein with IgA-kappa. Coagulation profile, urinalysis, and arterial blood gas were within the normal range. Pulmonary function test revealed small airway obstruction, which showed forced vital capacity (FVC) of 1.73 L (88.7% of predicted), forced expiratory volume in 1 second (FEV1) of 1.20 L (109.0% of predicted), FEV1/FVC of 69.3%, V50 of 1.04 L/sec, V25 of 0.21 L/sec, and V50/V25 of 4.95. She was diagnosed as Sjögren syndrome based on these findings.

**Figure 2:**
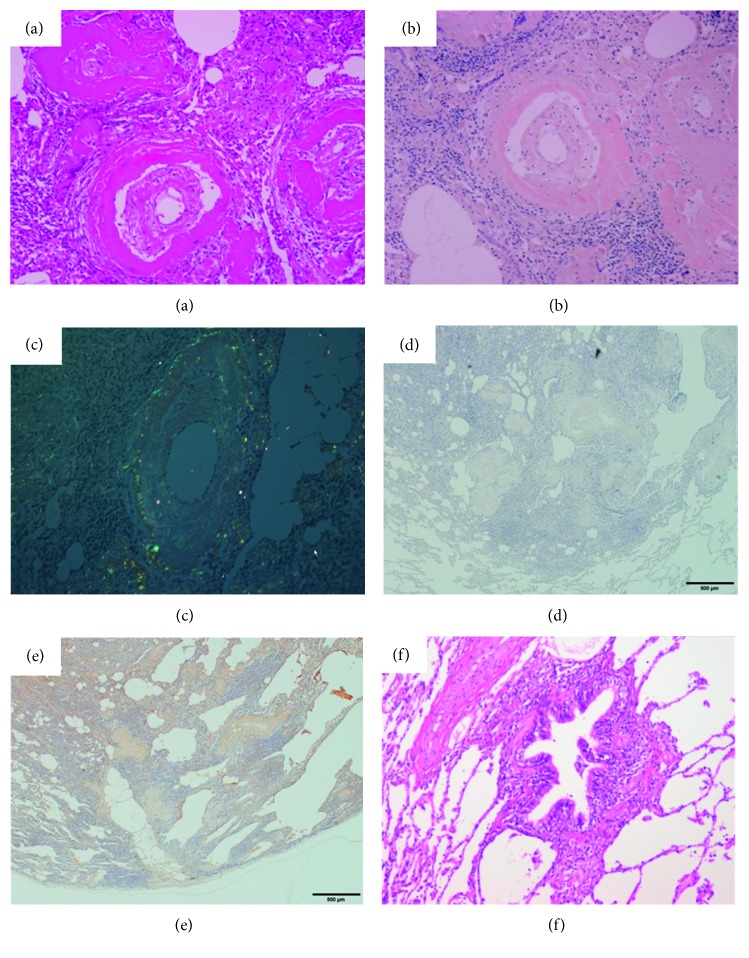
Pathologic findings of the surgical lung biopsy in a 72-year-old woman with Sjögren syndrome and pulmonary amyloidosis. (a) There is a homogeneous eosinophilic material in the vascular area (hematoxylin and eosin stain, ×100), which (b) was positive on Congo red stain (×100). (c) Polarizing microscopy shows apple green birefringence (×100). (d) The immunohistochemical staining with AA amyloid (×100) was negative. (e) The immunohistochemical staining with AL-kappa amyloid (×100) was positively stained. (f) There is infiltration of inflammatory cells in the peribronchial areas (hematoxylin and eosin stain, ×100).

Surgical lung biopsy was performed from the left lower lobe, which contained a nodule. The pathologic findings (Figure 2) revealed the presence of amyloid proteins that appeared as homogeneous eosinophilic materials on hematoxylin-eosin and stained on Congo red with apple green birefringence on polarized microscopy (data not shown). Congo red stain performed by standard and polarized light and immunohistochemical staining with AA amyloid and AL-kappa amyloid classified the amyloid protein as an AL-kappa. Moreover, there was peribronchiolar infiltration of inflammatory cells, mainly lymphocytes. Although we planned salivary gland biopsy, we could not obtain consent from the patient. She was finally diagnosed as Sjögren syndrome with lymphocytic bronchiolitis and pulmonary amyloidosis.

**Figure 3:**
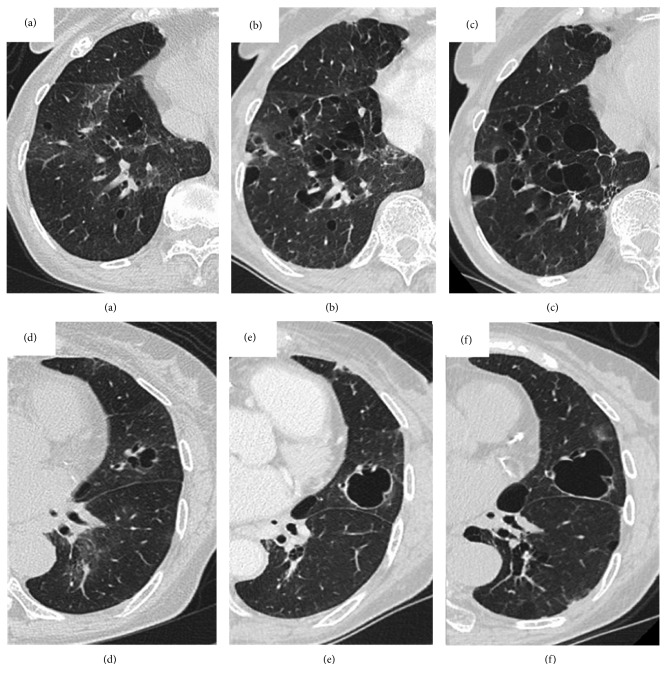
Surveillance chest computed tomography in a 72-year-old woman with Sjögren syndrome and pulmonary amyloidosis after 6 years of follow-up. The cystic lung lesions gradually increased in size and number after 6 years. (a–c) The right lower lobe and (d–f) the left upper and lower lobes are shown. (a) and (d) are images taken upon the time of diagnosis, (b) and (e) are images taken after 3 years, and (c) and (f) are images taken after 6 years.

Because of the absence of respiratory symptoms, she was followed by chest CT and pulmonary function test surveillance without treatment. There was no systemic involvement, including the heart, kidney, skin, and gastrointestinal tract during the follow-up period. After 6 years, the cystic lung lesions gradually enlarged and increased in number (Figure 3), but she remained to have no respiratory symptoms, no decrease in FEV1 (1.18 L), and no findings of lymphoproliferative disorder.

## 3. Discussion

The pulmonary manifestations of Sjögren syndrome include interstitial pneumonia, tracheobronchial disorders, and lymphoproliferative disorders [3]. Interstitial pneumonia and tracheobronchial disorders are common, whereas cystic lung disease and pulmonary amyloidosis are rare manifestations of Sjögren syndrome [1]. Meanwhile, amyloidosis is characterized by extracellular tissue deposition of abnormal amyloid proteins [4]. The World Health Organization classified amyloidosis on the basis of the structure of variable fibrillary proteins and subsequently divided it into primary or secondary disease [8]. The two major forms are AL amyloidosis and reactive amyloidosis or amyloid A (AA) amyloidosis, which is due to chronic inflammation. An AL comprises a clonal proliferation of plasma cells, and the fibrillary protein is a fragment of the Ig light chain [9]. On the other hand, AA is an acute-phase protein that is produced in response to systemic inflammation [6, 10]. All types of amyloid protein stain with Congo red and exhibit apple green birefringence on polarized microscopy [4]. Most cases of respiratory tract amyloidosis have been reported to have deposition of the AL-type of amyloid protein [5]. Sjögren syndrome is a chronic inflammatory disease and is also recognized as a lymphoproliferative disorder, similar to monoclonal gammopathy and AL amyloidosis. Lung involvement of amyloidosis in Sjögren syndrome has been reported as AL amyloidosis [11]. Amyloid deposition was considered to be related to the lymphoproliferative process seen in the lung.

Cystic lung disease can be associated with not only Sjögren syndrome with lymphocytic bronchiolitis [2] but also with pulmonary amyloidosis [12]. A previous report stated that among 21 patients with amyloid-associated cystic lung disease, collagen vascular disease (CVD) was present in 12 patients and absent in 9 patients [12]. The group with cystic amyloid and CVD had more women and normal pulmonary function test results than did the cystic amyloid group without CVD. In that report, the patients with amyloidosis without CVD had more obstructive pulmonary function than those with amyloidosis and CVD; this was consistent with the characteristics of the present case. Cystic formation has been suggested to be due to the presence of inflammatory cells or amyloid causing a check valve mechanism, which leads to bronchial obstruction [13]. In one study that analyzed and compared the high-resolution CT (HRCT) and histopathologic findings of amyloidosis in Sjögren syndrome, the cysts appeared to be located in the distal bronchioles that were narrowed by amyloid deposition [11]. In the present case, the cysts were located in the distal bronchioles on sequential HRCT findings (Figure 3). A previous study reported that 12 of 13 patients with cystic lung disease in Sjögren syndrome did not show disease progression after 4 years of follow-up [2]. Interestingly, the present case was observed to have gradual increase in the number and size of the lung cystic lesions for 6 years, which is a rare clinical course. This may indicate the natural progression of interstitial pneumonia to cystic lung disease.

The characteristic pulmonary function test of patients with Sjögren syndrome and cystic lung disease is often an obstructive pattern, which the present case likewise manifested [2]. This obstructive pattern on pulmonary function test is considered to suggest airway narrowing secondary to bronchiolitis. Notably, the FEV1 in the present case did not decline within 6 years of diagnosis.

Sjögren syndrome is associated with increased risk of non-Hodgkin lymphoma [1], the most common subtypes of which are mucosa-associated lymphoid tissue and marginal zone B-cell lymphoma. In fact, the prevalence of pulmonary lymphoma in patients with primary Sjögren syndrome was reported to be 1%-2% [14]. Physicians should also observe for the development of pulmonary lymphoma in these patients.

In conclusion, we reported a rare case of Sjögren syndrome complicated with pulmonary amyloidosis with cystic formation. Physicians should consider amyloidosis in the differential diagnosis of patients with cystic lung shadows and should proactively perform pathologic examination.
